# Targeting the Gut Microbiota in the Treatment of Type 2 Diabetes: Dietary Interventions, Microbial Preparations, and Fecal Transplantation

**DOI:** 10.2174/0115733998411102251127094217

**Published:** 2026-01-13

**Authors:** Lingling Ding, Zhiyuan Xi, Yuxi Zou, Shun Li, Daoyi Chen, Yuwei Liu, Junfang Zhao

**Affiliations:** 1 Department of Physiology, School of Medicine, Jiangsu University, Zhenjiang, P.R. China;; 2 Department of Microbiology, School of Medicine, Jiangsu University, Zhenjiang, P.R. China;; 3 Branch of the national clinical research center for metabolic disease, Wuhan, P.R. China;; 4 Department of Biliary and Pancreatic surgery, Tongji Hospital, Tongji Medical College, Huazhong University of Science and Technology, Wuhan, P.R. China

**Keywords:** Type 2 diabetes mellitus, gut microbiota, dietary intervention, metabolites, fecal microbiota transplantation, short-chain fatty acids (SCFAs)

## Abstract

Type 2 diabetes mellitus (T2DM) is the most prevalent metabolic disease worldwide, characterized by hyperglycemia and insulin resistance (IR). Its escalating global prevalence and the associated morbidity and mortality render it a major public health concern. Conventional glucose-lowering therapies frequently entail adverse effects, hypoglycaemia risk, and fail to arrest disease progression. Emerging evidence positions the gut microbiota as a central regulator of glucose homeostasis and insulin sensitivity, suggesting that gut microbiota might be a promising target for T2DM. This review synthesizes current knowledge of microbiota-driven mechanisms, particularly those of the gut microbiota and their metabolites, that precipitate or exacerbate T2DM. It then critically evaluates microbiota-targeted interventions (dietary modulation, probiotics, prebiotics, antibiotic therapy, and fecal microbiota transplantation) as emerging therapeutic or adjunctive strategies to restore glycaemic control by modulating the gut microbial ecosystem. While clinical validation is incomplete, targeting the gut microbiota represents a promising avenue for both prevention and treatment of T2DM.

## 1 INTRODUCTION

Type 2 diabetes mellitus (T2DM) is a chronic metabolic condition characterized by impaired insulin secretion from the pancreatic β cells and reduced insulin sensitivity in peripheral tissues [1]. Epidemiological data indicate an alarming annual increase in the prevalence of T2DM. Currently, an estimated 420 million individuals worldwide live with diabetes, more than 90% of whom have T2DM [2, 3]. The International Diabetes Federation (IDF) projects this number will rise to 640 million by 2040, solidifying T2DM as a critical global health challenge. Current pharmacotherapies (*e.g.*, metformin, insulin, SGLT2 inhibitors) form the cornerstone of T2DM management but are often associated with adverse effects, hypoglycemia risk, and an inability to halt disease progression or prevent complications in all patients, necessitating the exploration of complementary and alternative strategies [4].

Advances in scientific methodologies have revolutionized the study of microorganisms, leading to the recognition of the gut microbiota as a vital “virtual organ” fundamentally involved in the pathogenesis of chronic diseases, including cardiovascular disease, inflammatory bowel disease, cancer, and T2DM [5-8]. Among these chronic diseases, the relationship between the gut microbiota and T2DM has become a focal point of research. Growing evidence highlights how gut microbiota and their metabolites affect T2DM pathogenesis. Recently, most research has shown that T2DM patients exhibit gut dysbiosis, characterized by reduced abundance of butyrate-producing and sulfate-reducing bacteria [9-11], suggesting the essential role of the gut microbiome in T2DM. Thus, elucidating the crosstalk between the gut microbiota and T2DM may provide novel insights into the therapeutic approaches for T2DM.

This review will synthesize the latest evidence from both human clinical trials and animal models to comprehensively explore the role of gut microbiota in T2DM. Although current glucose-lowering drugs (*e.g.*, metformin, insulin) form the cornerstone of T2DM management, they may be associated with gastrointestinal side effects, hypoglycemia risk, and an inability to fully prevent disease progression or complications. Therefore, exploring novel therapeutic strategies targeting the gut microbiota is of significant clinical importance. We subsequently discuss advancements in gut microbiota-targeted therapeutic strategies for T2DM management, including dietary interventions, antibiotics, probiotics, prebiotics, and fecal microbiota transplantation (FMT).

## 2 LITERATURE SEARCH STRATEGY

A comprehensive literature search was conducted to identify relevant studies exploring the role of gut microbiota in the pathogenesis and treatment of T2DM. The search was performed using the following electronic databases: PubMed, Web of Science, and Scopus. The search timeframe extended from January 2010 to December 2025 to capture the most recent and relevant advancements in the field. The search terms included: 'type 2 diabetes mellitus', 'T2DM', 'gut microbiota', 'gut microbiome', 'microbiome', 'probiotics', 'prebiotics', 'fecal microbiota transplantation', 'dietary intervention', 'short-chain fatty acids', 'metabolites'. Inclusion criteria were: (1) studies investigating the association between gut microbiota and T2DM pathogenesis; (2) interventional studies evaluating microbiota-targeted strategies (diet, probiotics, FMT, *etc.*) for T2DM; (3) original research articles (including observational studies and randomized controlled trials) and high-quality reviews.

### 2.1 Gut Microbiota: An Overview

The human gastrointestinal tract hosts the body’s densest and most complex microbial ecosystem [12]. A healthy adult carries more than 100 trillion microorganisms, roughly equal to the number of human cells [13], accounting for approximately 0.3% of body weight [14]. The intestinal flora is composed of more than 1,000 microorganisms, primarily within the *Firmicutes*, *Bacteroidetes*, *Actinomycetes*, and *Proteobacteria* [15]. The gut microbiota is not innate but acquired postnatally, with initial colonization occurring through maternal and environmental exposure after birth [16]. Additionally, age, gender, medications, dietary habits, and trace elements also influence the activity of the gut microbiota [17-21].

Humans can maintain a harmonious coexistence with the intestinal microbiota. The host provides nutrients and a favorable environment for these microbes, while, in turn, the gut microbiota contributes to the regulation of glucose and lipid metabolism under normal conditions. It achieves this by breaking down dietary components, facilitating nutrient absorption, and producing metabolites, such as vitamins and short-chain fatty acids (SCFAs), that support host growth and metabolic health [22-24]. Conversely, gut microbiota imbalance (dysbiosis) is implicated in the pathogenesis of numerous metabolic and chronic diseases, including irritable bowel syndrome, radiation enteritis, polycystic ovarian syndrome, hypertension, T2DM, and chronic kidney disease [23, 25-30].

Dietary composition is a major environmental factor shaping the gut microbiota. High consumption of dietary fats and sugars, hallmarks of Western diets, is a significant contributor to intestinal dysbiosis and metabolic disorders like obesity and T2DM [31, 32]. Studies have reported that excess digested fat and high sugar intake are among the main factors contributing to the colonization of the gut by *Enterobacteriaceae* [33]. For instance, genotoxic *E. coli* may influence the stability of host cell DNA by producing *E. coli* that disrupts DNA double-strand integrity [34]. This suggests that the gut microbiota should be at the center of the new metabolic infection triad, comprising gut dysbiosis, metabolic disease, and gut bacterial infection [35-38], thereby serving as a promising therapeutic target for metabolic diseases, including T2DM.

### 2.2 Gut Microbiota in T2DM

Relevantly, gut microbiota plays a dual role in T2DM, acting as either a protective or pathogenic factor. Research has shown that individuals with T2DM exhibit gut microbial dysbiosis, for instance, higher levels of *Bifidobacterium*, *Streptococcus,* and *Prevotella* observed in Asian diabetic patients [39]. Besides, accumulating evidence demonstrates a robust inverse association between specific gut commensals, namely *Bifidobacterium*, *Bacteroides*, *Faecalibacterium*, *Akkermansia,* and *Roseburia*, and T2DM, implicating these taxa as potential protective factors against disease development [40]. As illustrated in Fig. (**1**), *Bifidobacterium* is the most frequently reported genus associated with protection against T2DM. Animal studies involving various *Bifidobacterium* species consistently demonstrate improved glucose tolerance [40]. *Bacteroides* is another beneficial genus, shown to positively influence glucose metabolism in both humans and animal models [41]. *Roseburia*, *Faecalibacterium*, and *Akkermansia* have also been negatively correlated with T2DM, supporting their protective roles [42, 43]. For example, a study reported that *Akkermansia* reversed diet-induced metabolic endotoxemia and related diseases in obese mice. This reversal resulted in weight loss, improved insulin sensitivity, reduced fasting hyperglycemia, and changes in gene expression regulating fat cells and lipid oxidation. However, an increase in Gram-negative bacteria can lead to systemic, low-grade chronic inflammation. For instance, excessive proliferation of *Proteobacteria* can cause intestinal leakage, loosening of tight junctions, and increased mucosal permeability. When LPS leaks into the bloodstream, it triggers oxidative stress and the release of pro-inflammatory cytokines, thereby contributing to the development of T2DM [44].

Studies have also identified alterations in *Lactobacillus* and *Clostridium* species in diabetic patients. Notably, *Lactobacillus* levels often correlate positively with fasting blood glucose (FBG) and glycated hemoglobin (HbA1c), whereas certain *Clostridium* species show negative correlations with HbA1c, FBG, C-peptide, insulin, and triglycerides [45], indicating distinct roles in T2DM progression. However, the role of specific taxa, such as *Lactobacillus*, remains controversial. While some studies report adverse associations, others demonstrate that specific strains (*e.g.*, *Lactobacillus casei*, *Lactobacillus rhamnosus GG*) can improve insulin sensitivity and glucose control [46]. These discrepancies likely arise from strain-specific effects, study population heterogeneity (diet, geography), and differences in T2DM duration and severity, emphasizing the need to define microbial function at the species or strain level.

Beyond reduced beneficial bacteria, T2DM patients often exhibit increased pathogenic species, such as *Clostridium hathewayi*, *Clostridium symbiosum*, and *Escherichia coli*, compared to healthy individuals, who typically harbor a greater abundance of butyrate-producing bacteria [47]. Collectively, these findings underscore the complex and sometimes contradictory interplay between gut microbiota composition and T2DM, offering crucial insights into its pathogenesis and highlighting potential therapeutic targets.

### 2.3 Impact of Gut Microbiota Metabolites on T2DM

The influence of the gut microbiota on T2DM extends beyond its cellular components to include its metabolic products, as illustrated in Fig. (**2**). A key function is the fermentation of non-digestible carbohydrates to produce short-chain fatty acids (SCFAs), primarily acetate (C2), propionate (C3), and butyrate (C4), which account for approximately 95% of total SCFAs [48-50]. Extensive preclinical and clinical evidence demonstrates that SCFAs can ameliorate T2DM by improving insulin resistance, promoting glucose homeostasis, and reducing fat accumulation and inflammation [51, 52].

For instance, C2 and C3 can stimulate lipogenesis through GPCR43 to reduce lipolysis and reduce plasma-free fatty acid levels [53]. Propionate also modulates gluconeogenesis through interactions with GPCR41 (FFAR3), GPCR43, and GPR109A (HCAR2) [54]. Butyrate, in addition to being a primary energy source for colonocytes, reduces lipolysis and inhibits NF-κB-mediated inflammatory signaling, thereby attenuating inflammation triggered by adipocyte-macrophage interactions [55].

Moreover, indole and its derivatives (*i.e.*, indole-3-propionic acid, IPA) are other microbiota metabolites that have been shown to have a beneficial effect on T2DM. Notably, indole can regulate the secretion of glucagon-like peptide-1 (GLP-1) from intestinal enteroendocrine L cells [56], thereby promoting insulin secretion and inhibiting glucagon secretion. Similarly, IPA has been found to improve insulin resistance, likely by preserving β-cell function [57].

In addition, bile acid (BA)-derived metabolites are another type of intestinal microbiota product associated with the pathology of T2DM [58]. Relatively, primary BAs can conjugate with glycine or taurine to form conjugated BAs, such as glycocholic acid (GCA) and tauroursodeoxycholic acid (TUDCA) [59, 60]. These conjugated BAs can be converted into secondary BAs (*i.e.*, lithocholic acid (LCA), deoxycholic acid (DCA), ursodeoxycholic acid (UDCA)) *via* deconjugation, dehydroxylation, and epimerization by gut microbes, such as *Bacteroides*, *Eubacterium,* and *Clostridium* [52, 61]. Studies indicated that BAs regulate glucose homeostasis by acting on two receptors, FXR (farnesoid X receptor) and TGR5 (G protein-coupled bile acid receptor 1). For instance, administering colesevelam (a BA sequestrant) to *ob/ob* mice suppressed FXR activity in intestinal L- cells, which in turn stimulated the production and secretion of GLP-1, leading to improved glycemic control. Relevantly, trimethylamine N-oxide (TMAO), derived from choline, carnitine, or betaine, metabolized by gut microbiota, has also been found to be associated with T2DM. For instance, a clinical study has demonstrated an elevated plasma TMAO concentration in T2DM individuals, which further elevates the risk of major adverse cardiac events and all-cause mortality, highlighting the pathologic role of TMAO in T2DM [62]. Thus, gut microbiota metabolites can exert either beneficial or detrimental influences on T2DM.

## 3 THERAPEUTIC TARGETING OF THE GUT MICROBIOTA IN T2DM

The gut microbiota presents a significant therapeutic potential for T2DM management. Evidence supports various microbiota-targeted interventions, including dietary modification, probiotic/prebiotic supplementation, antibiotic therapy, and fecal microbiota transplantation (FMT), as summarized in Fig. (**3**) [45].

### 3.1 Diet Interventions

Dietary changes can induce rapid shifts in the microbial community within 24 hours. Long-term dietary patterns are crucial for maintaining microbial diversity and abundance, and dietary modulation represents a powerful therapeutic strategy for T2DM [20, 63, 64]. As shown in Fig. (**4**), emerging studies have demonstrated that dietary interventions have the potential to exert therapeutic effects on T2DM, as discussed below [65-67].

#### 3.1.1 Intermittent Fasting (IF)

IF regimens positively impact metabolic health, potentially through changes in gut microbiota. IF improves T2DM symptoms, insulin sensitivity, glucose metabolism, and inflammatory responses [68-70]. In *db*/*db* mice, IF improved insulin signaling and cognitive function by rebuilding the gut microbiota and altering microbial metabolites [71]. In diet-induced obese mice, IF increased *Akkermansia muciniphila* abundance, reduced *Firmicutes* prevalence, and modulated bile acid metabolism, thereby ameliorating insulin resistance [72]. Similarly, IF reduced FBG, body weight, and insulin levels in STZ-HFD-induced T2DM mice [73].

#### 3.1.2 Dietary Fiber

Soluble dietary fiber (SDF) resists digestion but is readily fermented by gut bacteria, significantly modulating microbial composition and diversity. SDF fermentation produces SCFAs, which improve glucose and lipid parameters [74, 75]. Fiber intake increases *Bifidobacterium* abundance and reduces lipopolysaccharide (LPS) and total cholesterol levels [76]. A high-fiber diet enriched beneficial bacteria (*Lactobacillus*, *Bifidobacterium*, and *Akkermansia*), reduced pathogens (*Desulfovibrio*, *Klebsiella*), and improved glucose homeostasis, serum metabolism, and mood in T2DM patients [77].

#### 3.1.3 Mediterranean Diet

The Mediterranean diet, rich in whole grains, vegetables, fruits, olive oil, nuts, and fish, with moderate dairy and wine and limited red meat and sugar, is recommended for T2DM management [78-80]. High adherence is associated with a 19% reduced T2DM risk [81]. Its benefits for obesity and diabetes are partly attributed to promoting a health-favorable gut microbiota composition [82, 83], enhancing microbial metabolite production, improving intestinal barrier function, and reducing dysbiosis and permeability [84].

#### 3.1.4 Chinese Medical Nutrition Therapy (CMNT)

CMNT combines Medicine Food Homology (MFH) plants, whole grains, and intermittent energy restriction [79]. It exerts glucose-lowering effects in diabetic mice and patients, correlating with altered gut microbiota and improved β-cell function in *db*/*db* mice [79]. MFH plants contain bioactive compounds (polysaccharides, flavonoids, alkaloids) that stimulate insulin secretion and modulate gut microbiota [85, 86]. Whole grains, such as highland barley, improve glucose homeostasis [87] and enrich *Bifidobacterium* and *Akkermansia*, alleviating inflammation and improving glucose metabolism in diabetic mice [88].

#### 3.1.5 Low-Carbohydrate Diets (LCDs)

Low-carbohydrate diets (LCDs), often defined as <130 g of carbohydrates per day or <26% of total energy from carbohydrates [89], improve glycemic control and lipid metabolism in overweight or obese patients with T2DM [90]. Very-low-calorie diets can even induce diabetes remission [91, 92]. However, some LCDs, particularly high-protein, normocaloric versions, may reduce beneficial butyrate-producing bacteria, such as the Roseburia/E. rectale group [93], potentially offsetting certain SCFA-related anti-inflammatory effects. Thus, LCDs may exert part of their beneficial effects on T2DM through modulation of the gut microbiota, but their specific impact warrants careful consideration.

### 3.2 Probiotics and Prebiotics

Probiotics contribute to host health by synthesizing vitamins, aiding in food digestion, enhancing intestinal peristalsis, suppressing pathogenic bacteria, and detoxifying harmful compounds. Prebiotics, which are specifically metabolized by beneficial gut bacteria, are converted into health-promoting metabolites such as short-chain fatty acids (SCFAs) and serve as growth substrates for probiotics. In diabetic patients, disruptions in the gut microbiota lead to reduced levels of both probiotics and prebiotics, thus restoring this balance can help mitigate diabetic symptoms [94]. Supplementation with prebiotics and probiotics can serve as an effective preventive strategy alongside diet and exercise [95], improving the gut microenvironment, lowering intestinal endotoxin concentrations [96], and alleviating glucose intolerance and insulin resistance in diabetes [97].

For example, in a randomized controlled trial, probiotics were shown to reduce fasting blood glucose more effectively than placebo or no-intervention groups. Moreover, probiotics were effective in reducing serum cholesterol both in the short and long term, which also contributed to blood-glucose-lowering effects in patients with T2DM [98]. This effect appears to be more pronounced in participants with poorly controlled diabetes, particularly those not receiving insulin therapy [99]. Additionally, multiple *in vitro* and *in vivo* studies indicate that consumption of dairy products, such as yogurt, fermented milk, and cheese, rich in probiotic strains and prebiotic components, can reduce hyperglycemia, depressive behavior, obesity, oxidative stress, and insulin resistance in patients with diabetes [100].

### 3.3 Antibiotic Therapy

Beyond dietary interventions, antibiotic therapy has emerged as a potential strategy for managing T2DM. Studies have shown that norfloxacin and ampicillin were able to reduce blood glucose levels in diet-induced obese rats [101]. Mechanistically, these antibiotics regulate the activity of γ-aminobutyric acid (GABA) receptors on pancreatic α-cells, thereby suppressing glucagon secretion [95]. Furthermore, appropriate antibiotic use may help inhibit inflammatory responses and slow the progression of T2DM. Researchers have also reported that metformin, a drug commonly used to treat T2DM, not only modulates inflammatory responses but also influences the abundance of SCFA-producing gut microorganisms [102]. These SCFA-producing bacteria stimulate the secretion of glucagon-like peptide-1 (GLP-1), which alleviates T2DM by enhancing insulin secretion, improving glucose homeostasis, reducing inflammation, and increasing host satiety [103].

### 3.4 Fecal Transplantation

#### 3.4.1 Advantages of Fecal Microbiota Transplantation and Preparation Methods

Fecal microbial transplantation (FMT) is a therapeutic procedure that involves transferring an entire, intact, and stable intestinal microbial community from healthy donor feces to recipients with microbiota-associated disorders. This intervention aims to restore microbial homeostasis, correcting dysbiosis and ameliorating disease pathology [104]. The historical origins of FMT date back to the 4^th^ century in China, where physician Ge Hong pioneered the oral administration of human fecal suspensions, known as “yellow soup,” to treat severe diarrhea [105]. Unlike historical practices, modern FMT requires standardized donor screening and sample processing protocols. Nevertheless, the detailed methodology for preparing FMT varies considerably across studies. Typically, donor stool samples (approximately 100-150 g) are collected and mixed with sterile saline to achieve preliminary homogenization, forming a fecal slurry using a high-speed blender. Larger particles, fibers, and undigested food are then removed by filtration through a metal sieve. The resulting homogeneous fecal suspension is transferred into sterile syringes and should be administered within 6 hours of collection to ensure microbiota viability [104].

Compared with probiotic or prebiotic supplementation, which is generally safe and convenient but often produces modest and strain-dependent effects, FMT offers the distinct advantage of transferring the entire gut microbiota as a functional “organ.” Meta-analyses of randomized controlled trials (RCTs) reported that probiotic supplementation leads to statistically significant but modest reductions in HbA1c (typically −0.3% to −0.6%) and fasting blood glucose compared to placebo [106]. These effects are often strain-specific and generally transient, reversing after treatment cessation. In contrast, FMT represents a more comprehensive approach by aiming to reboot the entire gut microbial ecosystem. Evidence for its use in T2DM is still emerging from small pilot RCTs, with heterogeneous but promising results. Some studies report substantial improvements in insulin sensitivity and β-cell function that can persist for weeks after a single treatment [107].

The FMT procedure is considered highly safe, with a low incidence of adverse reactions. Unlike traditional organ transplants, it does not trigger immune rejection, making it a relatively low-risk intervention [108]. Currently, FMT is primarily employed for relapsed or refractory *Clostridium difficile* infections (CDI), where it has demonstrated therapeutic efficacy superior to antibiotics [109]. Additionally, FMT has shown potential as an effective intervention in several other conditions, including inflammatory bowel disease [110], irritable bowel syndrome [111], obesity [112], and T2DM [113].

#### 3.4.2 Fecal Microbiota Transplantation in T2DM

Studies have demonstrated that FMT can play a therapeutic role by attenuating the progression of metabolic syndrome and ameliorating pathological manifestations in T2DM [114]. When conventional hypoglycemic drugs are ineffective or intolerable, FMT emerges as a viable therapeutic alternative through modulation of the gut microbiota [115]. Clinical investigations by Vrieze *et al.* revealed that male participants with metabolic syndrome who received a six-week infusion of donor gut microbiota exhibited significant improvements in insulin sensitivity and reduced the risk of developing T2DM [116]. Furthermore, De Groot *et al.* reported that obese, insulin-resistant male recipients of fecal microbiota from metabolic syndrome donors showed reduced insulin sensitivity compared to those receiving transplants from healthy donors, establishing a clear link between microbiota composition and metabolic function [117]. Likewise, supportive evidence from a double-blind, randomized, controlled trial demonstrated that FMT affects glucose and lipid metabolism in men with metabolic syndrome. After six weeks of FMT treatment, the allograft group exhibited a significant increase in insulin sensitivity and fecal microbial diversity, whereas no such changes were observed in the autograft group [118]. Moreover, Ding *et al.* found that FMT induced faster gut microbiome remodeling than dietary interventions alone. Meanwhile, patients with T2DM exhibited additional improvements in blood glucose, blood pressure, lipids, and body mass index after FMT [119]. Furthermore, sequencing analyses revealed that T2DM-associated dysbiosis heightens intestinal permeability, fueling chronic low- grade inflammation and thus disease progression [95]. Relevantly, FMT has been shown to promote the growth of butyrate-producing bacteria, which have beneficial effects on metabolic diseases [120]. Recent studies have demonstrated that FMT significantly improves metabolic parameters in *db/db* mice. Specifically, FMT intervention resulted in marked reductions in fasting and postprandial blood glucose, total cholesterol, triglycerides, and low-density lipoprotein cholesterol. Concurrently, a significant elevation in high-density lipoprotein cholesterol (HDL-C) levels was observed [121], highlighting that FMT has an ameliorating effect on glucose and lipid metabolism. This indicates that modulating gut microbiota composition, metabolites, or immune responses can improve the condition in individuals with T2DM, demonstrating the therapeutic potential of the intestinal microbiota.

## CONCLUSION AND FUTURE PERSPECTIVES

In summary, the gut microbiota profoundly influences T2DM pathogenesis, making it a compelling therapeutic target. This review has synthesized evidence from human and animal studies, highlighting the critical connections between microbial communities, their metabolites, and host metabolism in T2DM. Dietary modulation, probiotics/prebiotics, and FMT represent promising strategies for managing T2DM by reshaping the gut ecosystem. Despite our efforts to conduct a comprehensive search, this review is subject to several potential biases. The most significant limitation is the predominance of preclinical studies (*in vitro* and animal models) in the current literature on this topic, compared to human clinical trials. While preclinical studies are indispensable for elucidating molecular mechanisms, this imbalance introduces a translational bias. Findings from animal models may not always be directly applicable to humans due to interspecies differences in gut microbiota composition, host physiology, and immune response. Consequently, the mechanistic pathways discussed in this review, although promising, should be interpreted with caution and await further validation in human studies.

For future studies, there are also many challenges. The composition and function of the gut microbiome vary greatly between individuals, shaped by a combination of genetic background, dietary patterns, environmental exposures, and medication history. This complexity suggests that a universal “all-in-one” probiotic strategy may be difficult to achieve, and that future development urgently needs to move toward personalized, precise intervention models. Moreover, live biotherapeutic products (LBPs), including FMT, carry a potential risk of transmitting unknown pathogens, and there have been reports of serious infections associated with their use. Therefore, all LBPs must undergo rigorous safety evaluations, and comprehensive regulatory frameworks should be established to ensure patient safety. The mechanisms of action for the vast majority of microbiota-based interventions remain at the hypothesis stage. Research needs to extend beyond macro-level descriptions of community structure and investigate the specific functional bacteria, key active molecules, and corresponding host signaling pathways responsible for their effects, in other words, advancing from the level of “community ecology” to detailed “molecular mechanisms.” Lastly, despite emerging trials, microbiota-targeted prevention and treatment of T2DM remain constrained by (i) scarce long-term, placebo- controlled prevention studies on at-risk cohorts, (ii) predominant use of surrogate glycaemic end-points without hard-outcome validation, and (iii) a lack of direct comparative dose-response data for identical interventions in pre-diabetes versus established diabetes. Altogether, despite the complexity of the microbiome, these challenges will determine whether microbiota-targeted strategies can become precision tools for T2DM prevention and treatment.

## Figures and Tables

**Fig. (1):**
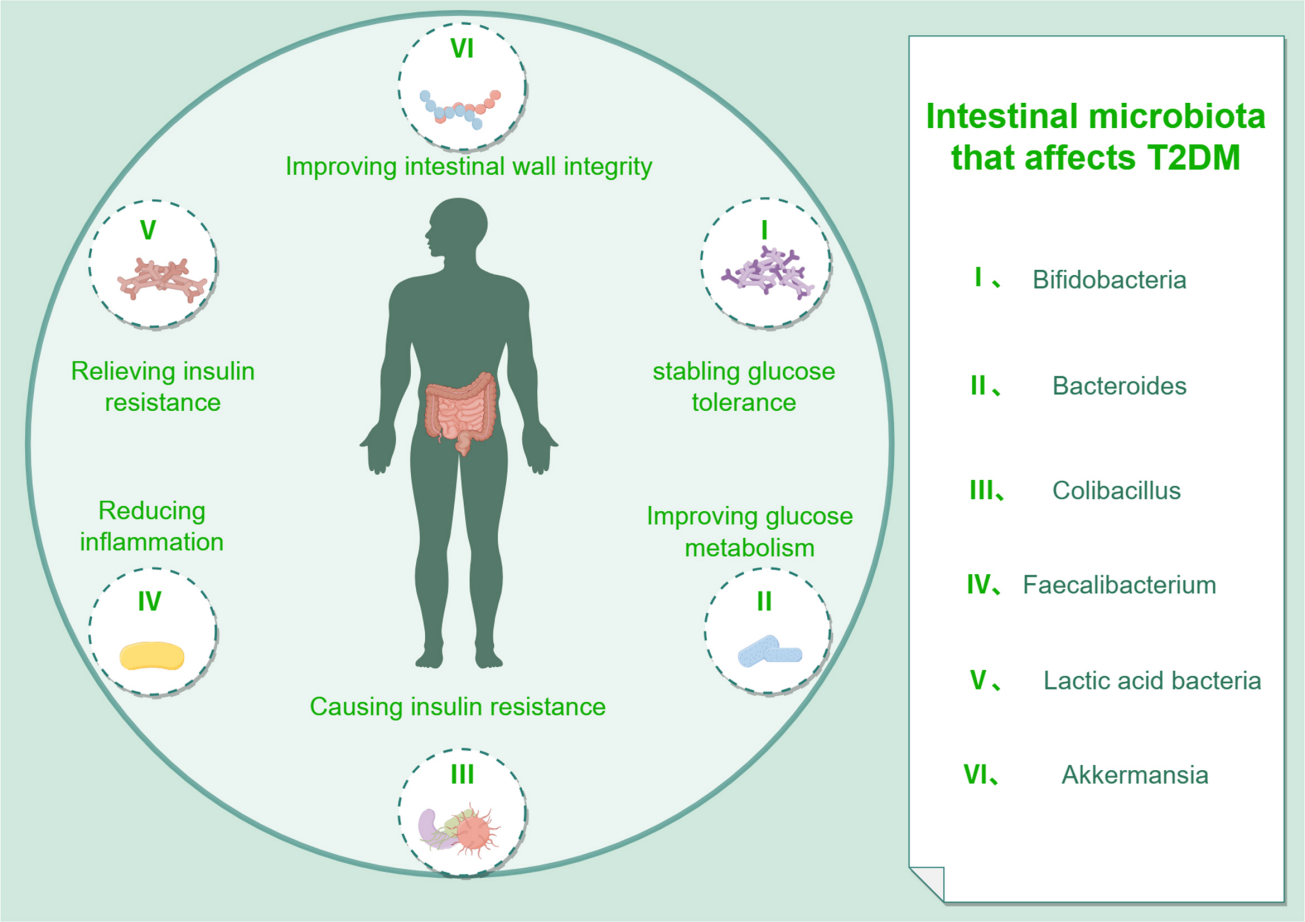
Intestinal microbiota and its effects on T2DM. The gut microbiota exerts diverse effects on T2DM. *Bifidobacterium* has been shown to improve glucose tolerance in several studies. *Bacteroides* play a beneficial role in glucose metabolism in both humans and animal models. *Escherichia coli* (Colibacillus) can affect host cell DNA stability, potentially causing DNA double-strand breaks and activating DNA damage checkpoint pathways, which may lead to cell cycle arrest, cell death, and insulin resistance. *Faecalibacterium* reduces intestinal inflammation, while lactic acid bacteria can lower insulin resistance. *Akkermansia* enhances intestinal barrier integrity and improves the overall gut environment.

**Fig. (2):**
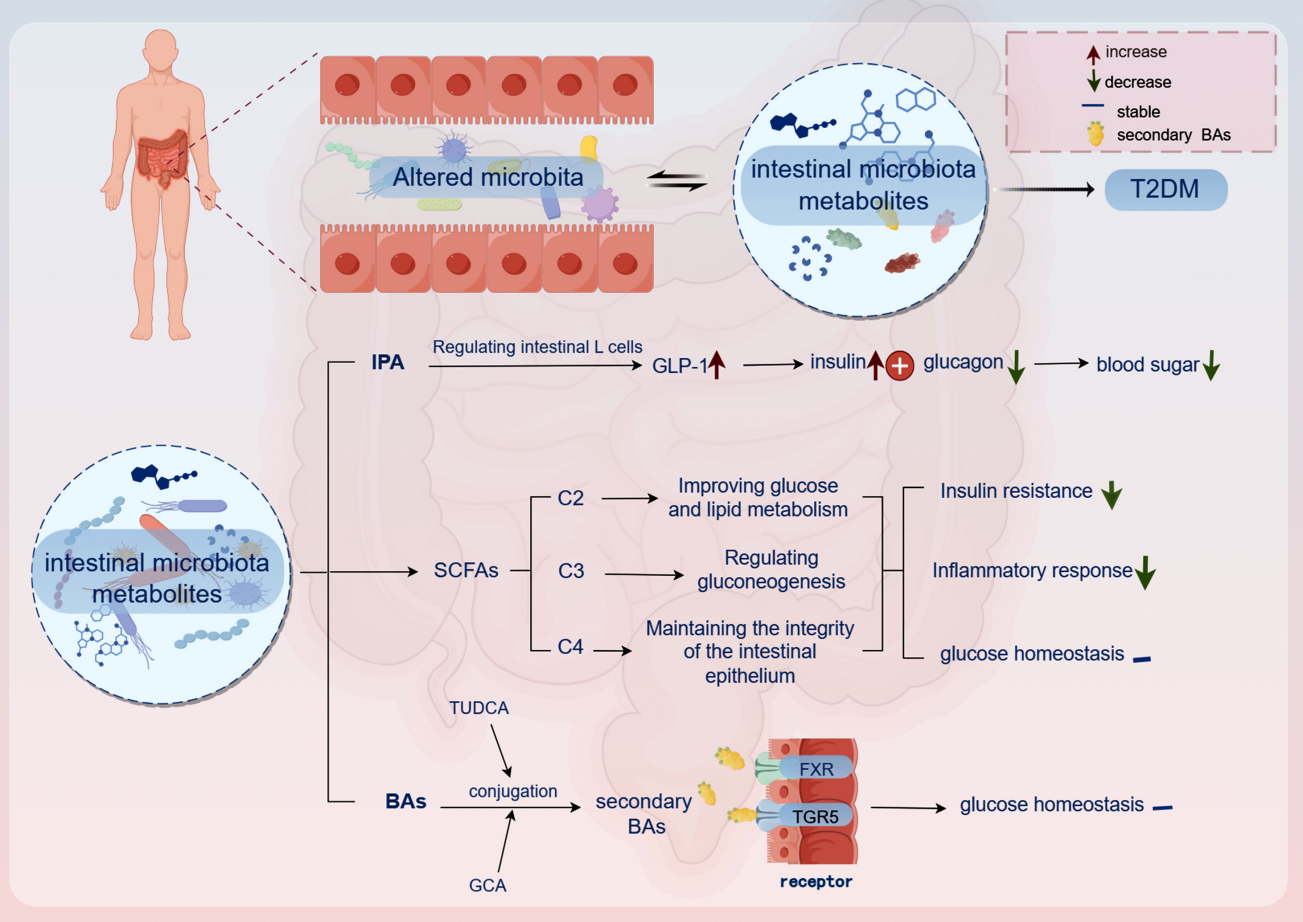
Effects of gut microbiota metabolites on T2DM. Three main types of intestinal metabolites influence T2DM. Indolepropionic acid (IPA) regulates intestinal L cells to stimulate GLP-1 secretion, thereby increasing insulin secretion, decreasing glucagon secretion, and lowering blood glucose. Short-chain fatty acids (SCFAs) exert multiple effects: acetate (C2) promotes lipogenesis, inhibits lipolysis, and improves glucose and lipid metabolism; propionate (C3) acts through GPR109A, GPR43, and GPR41 to regulate gluconeogenesis; and butyrate (C4) maintains intestinal epithelial integrity and inhibits inflammatory responses. Overall, SCFAs reduce insulin resistance, alleviate inflammation, and help maintain glucose homeostasis. Bile acids (BAs) are converted from primary BAs conjugated with TUDCA and GCA into secondary BAs, which bind to FXR and TGR5 receptors to support glucose homeostasis.

**Fig. (3):**
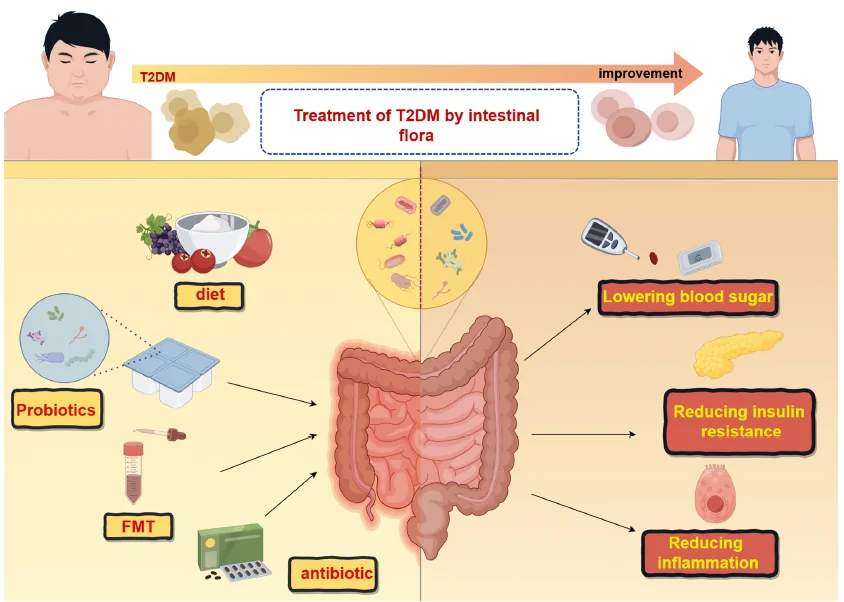
Diet interventions exert beneficial effects on T2DM by rebuilding the intestinal microbiota and improving microbial metabolite profiles. Approaches, such as intermittent fasting, dietary fiber intake, the Mediterranean diet, Chinese medical nutrition therapy, and low-calorie diets, can help lower blood glucose by enhancing insulin sensitivity, increasing SCFA production, supplying essential nutrients, regulating qi and blood, and reducing inflammation, respectively.

**Fig. (4):**
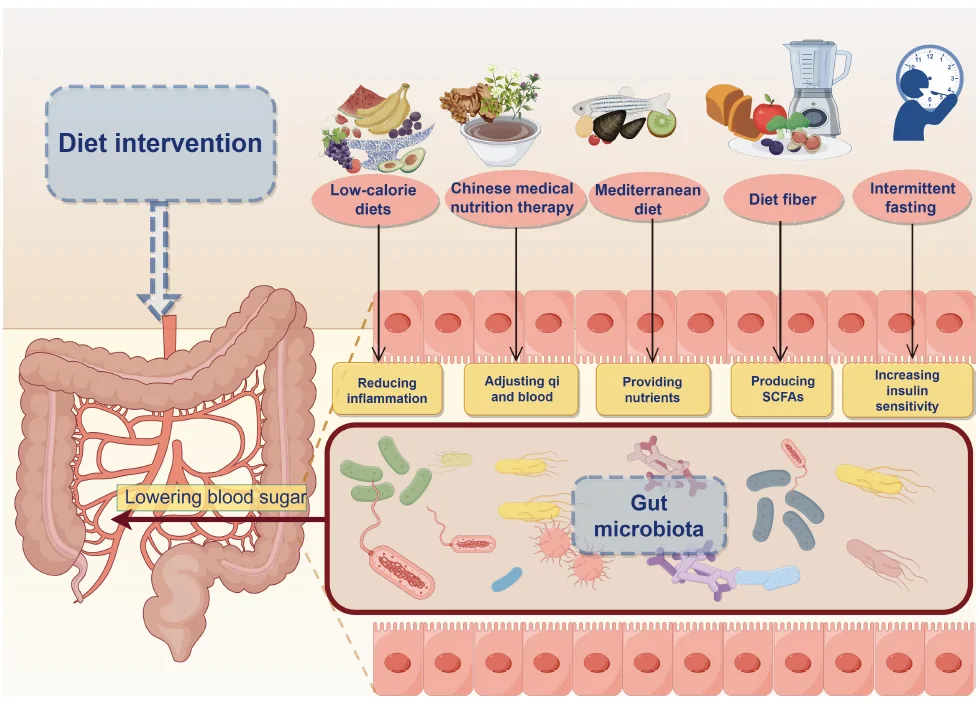
Application of intestinal microbiota in the treatment of T2DM. Studies have shown that dietary interventions, probiotic and prebiotic supplementation, antibiotic therapy, and fecal microbiota transplantation can improve glucose metabolism, enhance insulin sensitivity, and reduce inflammation in patients with T2DM by modulating the diversity and abundance of the intestinal microbiota.

## LIST OF ABBREVIATIONS

T2DM: Type 2 Diabetes Mellitus
FBG: Fasting Blood Glucose
GCA: Glycocholic Acid
LCA: Lithocholic Acid
UDCA: Ursodeoxycholic Acid
CMNT: Chinese Medical Nutrition Therapy
LCDs: Low-Carbohydrate Diets
FMT: Fecal Microbial Transplantation
HDL-C: High-density Lipoprotein Cholesterol
LBPs: Live Biotherapeutic Products
